# Data set on the effectiveness of flip teaching on engineering students' performance in the physics lab compared to Traditional Methodology

**DOI:** 10.1016/j.dib.2019.104915

**Published:** 2019-11-30

**Authors:** José A. Gómez-Tejedor, Ana Vidaurre, Isabel Tort-Ausina, José Molina Mateo, María-Antonia Serrano, José M. Meseguer-Dueñas, Rosa M. Martínez Sala, Susana Quiles, Jaime Riera

**Affiliations:** a Departamento de Física Aplicada, ETS de Ingeniería del Diseño, Universitat Politècnica de València, Camino de Vera, s/n, 46022, Valencia, Spain; b Departamento de Física Aplicada, ETS de Ingeniería de Edificación, Universitat Politècnica de València, Camino de Vera, s/n, 46022, Valencia, Spain

**Keywords:** Adult learning, Distributed learning environments, Improving classroom teaching, Multimedia/hypermedia systems, Teaching/learning strategies

## Abstract

This paper shows the data of the Flip Teaching and Traditional Methodology on the laboratory practice in two subjects, Physics and Electricity, of a technical degree. The laboratory and final grades of these subjects were shown in four consecutive years. The characteristics of all four years were quite similar, except that the Traditional teaching Methodology (TM) was used in two, while Flip Teaching methodology (FT) was applied in the other two.

For further discussion, please refer to the scientific article entitled “Effectiveness of flip teaching on engineering students' performance in the physics lab” [1]. Additional segmentation data in three levels are presented in this data in brief paper.

**Table:** Specifications Table

Subject	Education
Specific subject area	Physics Laboratory
Type of data	Tables and graphics
How data were acquired	After anonymization, grades were obtained from the courses in which the authors taught these subjects during the academic years studied.
Data format	Raw
Parameters for data collection	Data were collected at the end of the academic year. Any personal and gender data was deleted.
Description of data collection	The data was directly extracted from the University's grade management application
Data source location	Escuela Técnica Superior de Ingeniería del Diseño, Universitat Politècnica de València, Valencia (Spain)
Data accessibility	With the article and repository Mendeley Data: https://data.mendeley.com/datasets/68mt8gms4j/2
Related research article	José A. Gómez-Tejedor, Ana Vidaurre, Isabel Tort-Ausina, José Molina Mateo, María-Antonia Serrano, José M. Meseguer-Dueñas, Rosa M. Martínez Sala, Susana Quiles, Jaime Riera Effectiveness of flip teaching on engineering students' performance in the physics lab Computer and education, 144: 103708 (2020) DOI: 10.1016/j.compedu.2019.103708

**Table:** 

**Value of the Data** • These data are useful to deepen the students' performance in the physics lab for students who follow a Traditional Methodology and those who follow Flip Teaching. • Data on students' performance is useful to understand differences between Flip Teaching and Traditional Methodology. • These data can be used to make new analysis to compare students' performance following Flip Teaching and Traditional Methodology. • So far, there is no available data in the literature to compare Flip Teaching and Traditional Methodology in the Physics lab.

## 1 Data

The sample in this data paper was formed by 1233 students enrolled from 2013 to 2017 who completed the subjects of Physics (Phys) and Electricity (Elec) following two different methodologies: Traditional Methodology (TM) and Flip Teaching Methodology (FT) [1]. The previous grade that gave access to the university was very similar during all these years, between 6 and 7 (out of 10). The sample characteristics, the method used each year and course (TM or FT), the number of enrolled students, and the number of students that completed the course are summarized in Table 1 of reference [1], and more detailed information about the methodology is given in Ref. [1].

**Table 1:** Number of students (N), mean grade, standard deviation and cut-off grade between tercile 1 and 2 (T_1_/T_2_) and between tercile 2 and 3 (T_2_/T_3_) for the two subjects and the two methodologies: Physics Traditional Methodology (Phys TM), Physics Flip Teaching (Phys FT), Electricity Traditional Methodology (Elec TM), and Electricity Flip Teaching (Elec FT).

	Phys TM	Phys FT	Elec TM	Elec FT
N	305	299	315	314
Mean grade	7.05	7.32	5.60	6.20
Std. Deviation	1.42	1.24	1.60	1.38
Tercile 1/2 (T_1_/T_2_)	7.80	7.80	6.10	6.70
Tercile 2/3 (T_2_/T_3_)	6.50	7.00	5.09	5.50

The anonymized raw data with the individual grades in the laboratory and final grade in the courses and academic years of reference [1] are available through the Mendeley Data repository at https://data.mendeley.com/datasets/68mt8gms4j/2. The data is organized in columns where the laboratory and final grade is shown for the two courses and four academic years analysed. Every column header indicates the methodology used, the academic year and the course (Phys for Physics and Elec for Electricity). The “Experimental Design, Materials, and Methods” section explains in more detail how the grades are obtained.

In Ref. [1] the data was segmented in two groups (high and low performance levels) according to the median of the total grade for the course. In the present paper, the data was segmented in three groups: tercile 1 (*T*_1_), tercile 2 (*T*_2_) and tercile 3 (*T*_3_) according also to the median of the total grade for the course. In Table 1 is presented the number of students for the two subjects and the two methodologies, their mean grade with its standard deviation, and the cut-off grade between tercile 1 and tercile 2 (denoted as *T*_*1*_*/T*_*2*_) and between tercile 2 and tercile 3 (*T*_*2*_*/T*_*3*_).

### 1.1 Data grouped by methodology

First of all, we present the data grouped by the methodology used, independent of the subject, so joining the Phys TM and Elec TM to make the TM group (Traditional Methodology group) and Phys FT and Elec FT to make the FT group (Flip Teaching Methodology group). In Table 2 is shown the number of students following each methodology and the number of students in each tercile.

**Table 2:** Number of students following the Traditional Methodology (TM) and the Flip Teaching Methodology (FT), and number of students in every tercile (T_1_, T_2_ and T_3_).

		N
Method	TM	620
	FT	613
Tercile	T_1_	412
	T_2_	412
	T_3_	409

Table 3 shows the two-way ANOVA test where it is seen that the laboratory grade depends on the tercile, the methodology and the interaction between both of them.

**Table 3:** ANOVA analysis, where SG stands for the subgroup grade, M stands for methodology, SS stands for sum of squares, DF stands for the degrees of freedom.

	Source	SS	DF	F	p
Physics & Electricity	SG	611.27	2	162.73	<0.001
	M	34.48	1	18.36	<0.001
	(SG)*(M)	13.42	2	3.57	0.028

**Fig. 1:**
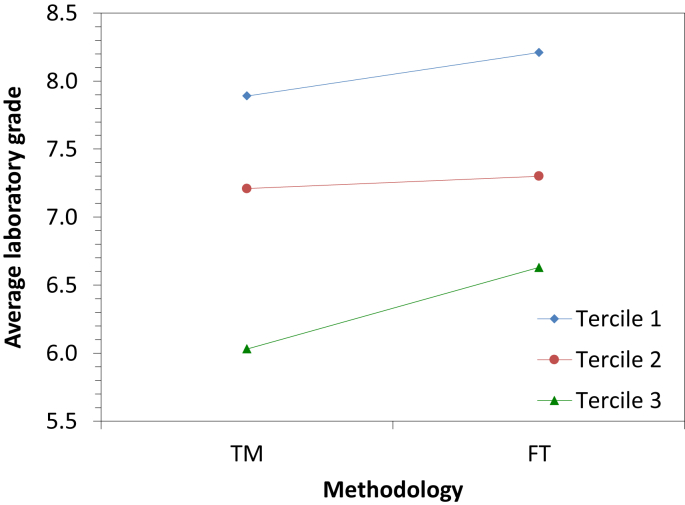
Average laboratory grade according to the methodology for the three terciles.

**Fig. 2:**
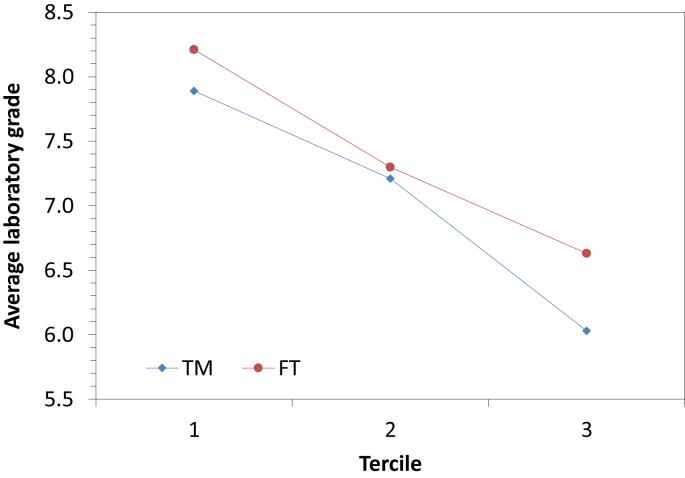
Average laboratory grade according to the tercile for the two methodologies.

Fig. 1 shows the average laboratory grade vs the methodology followed by the students for the three terciles of the data, and Fig. 2 the average laboratory grade vs the tercile for the two methodologies.

### 1.2 Data for physics

Now, we present the data grouped by the methodology (TM and FT) for the subject of physics. In Table 4 is shown the number of students following each methodology and the number of students in each tercile in the subject of physics.

**Table 4:** Number of students following the Traditional Methodology (TM) and the Flip Teaching Methodology (FT), and number of students in every tercile (T_1_, T_2_ and T_3_).

		N
Method	TM	305
	FT	299
Tercile	T_1_	202
	T_2_	202
	T_3_	200

Table 5 shows the two-way ANOVA test in the subject of physics.

**Table 5:** ANOVA analysis, where SG stands for the subgroup grade, M stands for methodology, SS stands for sum of squares, DF stands for the degrees of freedom.

	Source	SS	DF	F	p
Physics	SG	292.09	2	79.21	<0.001
	M	14.07	1	7.63	<0.001
	(SG)*(M)	6.57	2	1.78	0.17

**Fig. 3:**
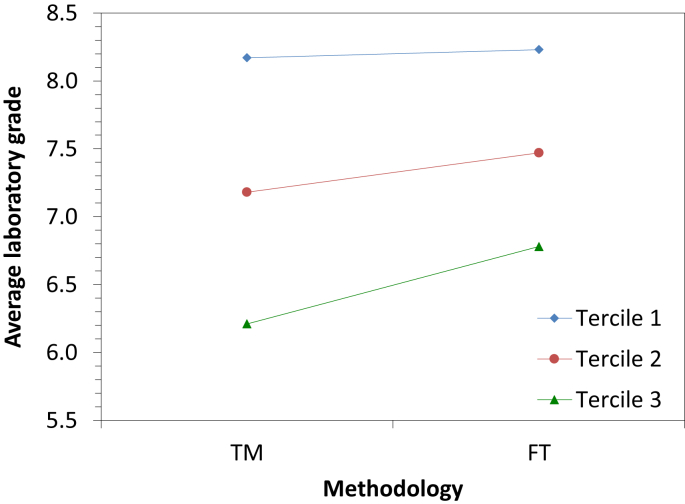
Average laboratory grade according to the methodology for the three terciles in the subject of physics.

**Fig. 4:**
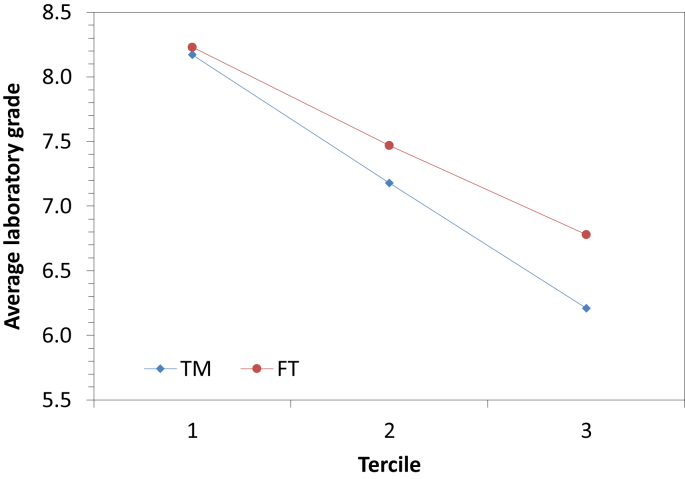
Average laboratory grade according to the tercile for the two methodologies in the subject of physics.

Fig. 3 shows the average laboratory grade vs the methodology followed by the students for the three terciles of the data, and Fig. 4 the average laboratory grade vs the tercile for the two methodologies, in the subject of physics.

### 1.3 Data for electricity

Finally, we present the data grouped by the methodology (TM and FT) for the subject of electricity. In Table 6 is shown the number of students following each methodology and the number of students in each tercile in the subject of electricity.

**Table 6:** Number of students following the Traditional Methodology (TM) and the Flip Teaching Methodology (FT), and number of students in every tercile (T_1_, T_2_ and T_3_).

		N
Method	TM	315
	FT	314
Tercile	T_1_	210
	T_2_	210
	T_3_	209

Table 7 shows the two-way ANOVA test for the subject of electricity.

**Table 7:** ANOVA analysis, where SG stands for the subgroup grade, M stands for methodology, SS stands for sum of squares, DF stands for the degrees of freedom.

	Source	SS	DF	F	p
Electricity	SG	321.35	2	85.59	<0.001
	M	20.84	1	11.10	<0.001
	(SG)*(M)	17.07	2	4.61	0.01

**Fig. 5:**
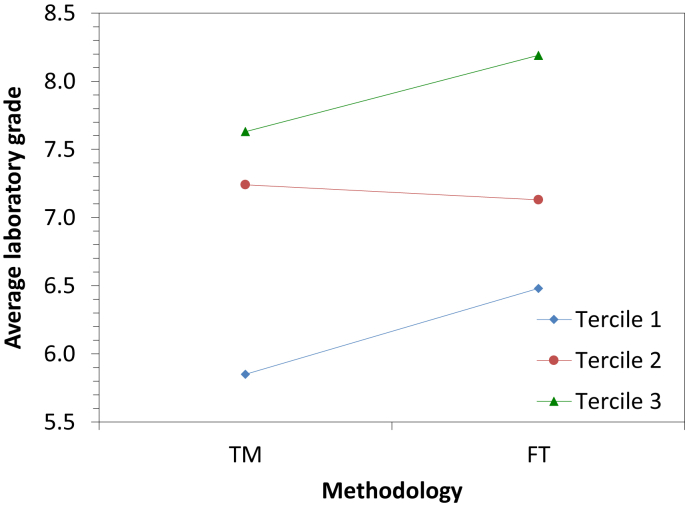
Average laboratory grade according to the methodology for the three terciles in the subject of electricity.

**Fig. 6:**
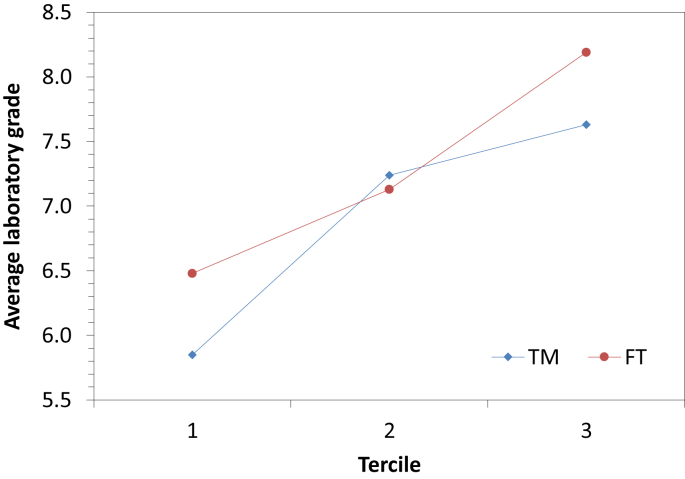
Average laboratory grade according to the tercile for the two methodologies in the subject of electricity.

Fig. 5 shows the average laboratory grade vs the methodology followed by the students for the three terciles of the data, and Fig. 6 the average laboratory grade vs the tercile for the two methodologies, in the subject of electricity.

## 2 Experimental Design, materials, and methods

The lab sessions were organized in the four years as follows: before the session the necessary learning material (several PDF documents and a video for the FT students) was made available to students through the *PoliformaT* online teaching platform. The documents and video explained the purpose and the procedure of the experience. An example of the video introducing the free fall practice can be seen at reference [2].

The main difference between the two method was that TM involved the teacher explaining the theoretical contents involved and lab procedure before the session, while FT involved the students studying the material in advance (reading the documents and watching videos). They were then expected to start the experience after any doubts had been answered.

Lab sessions were based on teamwork. After continuous teacher supervision, the students worked in groups of six. Each group then had ten days to upload a report. The lab report was graded using the rubric supplied in the supplementary data of reference [1], that was the same for all the groups in this paper.

To obtain the final grade, several items were assessed, with different weights: Traditional exam (70% of the grade), homework and classroom activities (10%) and lab report (20%).
